# Is endoscopic sinus surgery sufficient to modify the evolution of adult AERD? Aspirin desensitization as a maintenance factor: systematic review

**DOI:** 10.3389/falgy.2023.1250178

**Published:** 2023-09-08

**Authors:** Diego M. Conti, Eduardo J. Correa, Glenis K. Scadding

**Affiliations:** ^1^ The European Forum for Research and Education in Allergy and Airway Diseases Scientific Expert Team Members, Brussels, Belgium; ^2^ Otolaryngology Department, Hospital Comarcal de la Línea de La Concepción, Cádiz, Spain; ^3^ Department of Allergy & Rhinology, Royal National ENT Hospital, London, United Kingdom; ^4^ Division of Immunity and Infection, University College, London, United Kingdom

**Keywords:** AERD, N-ERD, ESS, FESS, sinus surgery, ATAD, aspirin desensitization, CRSwNP

## Abstract

**Introduction:**

Aspirin desensitization (AD) and aspirin therapy after desensitization (ATAD) are therapeutic interventions for patients with aspirin-exacerbated respiratory disease (AERD). Our aim is to investigate whether its addition to endoscopic sinus surgery (ESS) improves the overall prognosis of the disease.

**Methods:**

A systematic review of the current literature including adult patients with a positive diagnosis of AERD undergoing endoscopic sinus surgery (ESS) in the context or in absence of upper airway comorbidity, prior to AD + ATAD.

**Conclusion:**

This review concludes that the surgical approach is beneficial in AERD, but its effects are short—lived. Surgery should be considered initially with subsequent AD + ATAD in AERD patients, due to the sustained improvement achieved compared to those receiving ESS alone.

## Introduction

Non-steroidal anti-inflammatory drug (NSAID)-exacerbated respiratory disease (N-ERD), also known as Samter's triad or aspirin-exacerbated respiratory disease (AERD), is a chronic eosinophilic, inflammatory condition of the upper and lower airway tract that occurs in patients with Chronic Rhinosinusitis with Nasal Polyps (CRSwNP), asthma and hypersensitivity to COX-1 inhibiting drugs (1).

The disorder affects 0.6%–2.5% of the general population, 7% of patients with asthma and 15% of patients with severe asthma (2) and it is estimated that AERD will occur in around 40% of asthmatic patients with CRSwNP (3). The prevalence of aspirin (ASA) sensitivity is found in 16% of CRS cases, 28.3% in CRS without nasal polyps (CRSsNP) cases, and 38.5% in CRSwNP and asthma cases (4) and its peak onset occurs in the third and fourth decades of life (5).

CRS results in significant functional, emotional, and quality of life impairment with several associated comorbidities, some of them directly associated to its inflammatory nature and the concept of united airway such as allergic rhinitis or asthma, and others indirectly related such as diabetes, glaucoma, or obstructive sleep apnoea (6–8). AERD is one CRS phenotype that is less responsive to medical and/or surgical therapy compared to other phenotypes (9, 10). In addition, previous studies have found that AERD patients have objectively worse disease burden, poorer quality of life, and higher financial costs compared to their CRS counterparts (11).

Oral aspirin desensitization has demonstrated to be a safe and effective tool for the management of AERD (12) and has been shown to improve sinonasal outcomes measured by quality of life and need for revision surgery in multiple studies (13, 14).

On the other hand, Endoscopic Sinus Surgery (ESS) is key in the management of CRSwNP (15) in AERD and is primarily aimed at reducing tissue load and optimizing local medical treatment outcomes. However, the recurrence of nasal polyps after surgery is more frequent in AERD compared to CRSwNP patients without it (16) given a more extensive and aggressive sinonasal disease and higher rates of sinus surgery, and their polyps are more recalcitrant to both medical and surgical treatments (17, 18).

Typically, ESS is performed 2–4 weeks prior to starting aspirin desensitization and therapy to minimize the risk of polyp regrowth during the interval period and has been shown to be effective at controlling disease burden and symptoms (1, 2, 4). However, regardless of ESS technique and timing, aspirin desensitization followed by daily aspirin provides therapeutic benefits to most patients with AERD, including improved asthma control, decreased need for oral steroids and reduced nasal recurrence polyp (2).

This systematic review aims to investigate if endoscopic sinus surgery followed by AD + ATAD modifies the outcome, response or quality of life in adult patients with AERD and their comorbidities, compared to ESS alone (19, 20).

## Methods

We conducted a systematic review following the PRISMA guidelines (21), and registered in PROSPERO under the protocol number CRD42023431414. Inclusion criteria were defined as adult patients with a positive diagnosis of AERD, undergoing endoscopic sinus surgery (ESS) as single treatment in the context or in absence of upper airway comorbidity, prior to AD + ATAD. Exclusion criteria are shown in Table 1.

**Table 1:** Inclusion/exclusion criteria.

Inclusion criteria	Exclusion criteria
Patients older than 18 years	Patients younger than 18 years
AERD with or without comorbidities	No AERD
ESS (with or without AD + ATAD)	Biologics
Papers 2016–2023	Older papers
Prospective and retrospective studies, reviews and position papers	Case study descriptions

The search was conducted between April and May 2023, in PubMed, Scopus, Cochrane and Web of Science databases and designed using key Medical Subject Headings (MeSH) terms as referenced in PubMed Medical Subject Headings (Table 2).

**Table 2:** Mesh terms.

MeSH terms
AERD, N-ERD, NSAID, sinus surgery, ESS, FESS, ATAD
Aspirin, aspirin desensitization, aspirin challenge, CRS, CRSwNP
Asthma, guideline, consensus, position, statement, systematic review,
Assessment, evaluation and recommendations

Two researchers independently reviewed the titles and abstracts using Rayyan webpage (22, 23), including papers that might meet the inclusion criteria, identified whether each item was assessed and whether the final conclusions had strict relation with the objective of this work, and discarded those that did not fulfil it (Table 3). In case of conflict, a third researcher solved it. In order to evaluate each manuscript systematically, we designed a questionnaire to be fulfilled by the authors. The risk of bias and quality assessment was made following the ROBINS-I (“Risk Of Bias In Non-randomized Studies—of Interventions”) tool from Cochrane® (24).

**Table 3:** Systematic questionnaire to evaluate manuscripts.

Systematic questionnaire to evaluate and select manuscripts
Is this a prospective or retrospective study, a review or a position paper?
Does it include patients older than 18 years?
AERD diagnose was confirmed?
Was ESS performed as single treatment or prior to AD + ATAD?
Is the surgical extension declared?
Is the patient group followed up?
Is the surgical recurrence declared?
Are the success or failure rates declared?
Do the patients refer receive biologics at any point of their therapeutic schema?
Is SNOT-22 used?
What is the impact of this therapeutic intervention on the patient's comorbidities and/or quality of life?
Is the conclusion relevant to our review?

## Results

The treatment regimens presently available for the therapeutic management of patients with AERD are varied, as exemplified in Table 4. Therefore, we have selected to concentrate exclusively on the selection criteria discussed above, since they align with the aim of our review.

**Table 4:** Management of patients with AERD.

Management of patients with AERD (6, 13, 15)
1. Management of NSAID hypersensitivity a. Strict avoidance of the culprit drug and cross-reactive drugs b. Patient education c. Tolerance tests with alternative NSAID should be performed in the office before the drug is prescribed d. Alcohol avoidance should be advised to AERD patients e. Written information, including lists of potentially cross-reactive and alternative safe medications, should be always provided to AERD patients f. Patients should carry with them information about their drug hypersensitivity 2. Management of asthma a. The management for the AERD patient should be individualized; however, the severity of asthma should be assessed early in the disease course and considered in treatment decisions. b. Combination therapy with inhaled corticosteroid and long acting beta-2 agonists is sufficient to control asthma in the majority of AERD patients; short courses of oral corticosteroids may be needed for exacerbations c. Patient education re asthma control and rescue therapy 3. Management of chronic rhinosinusitis a. According to current guidelines, medical treatment of CRS should be based on topical corticosteroids with dosing adjusted to the severity of symptoms b. Short courses of oral steroids (2–3 weeks) may be needed to control severe CRS symptoms and to improve the quality of life c. Patient education d. Nasal saline irrigation, both isotonic and hypertonic, as well as short-term (before surgery) and long-term (after surgery) antibiotics may help to alleviate nasal symptoms e. Sinonasal surgery (polypectomy, functional endoscopic sinus surgery, and/or ethmoidectomy) is reserved for patients with severe or uncontrolled symptoms and for those with inadequate improvement despite intranasal and oral steroid therapy 4. Aspirin treatment after desensitization a. Desensitization procedure can be performed in both outpatient and hospital setting and should be supervised by experienced physician b. In the majority of AERD patients, ATAD is associated with a decrease in CRS symptoms, decrease in intranasal corticosteroid use, reduction in recurrence of nasal polyps, and decrease in the need for revision surgery c. In a subset of AERD patients, ATAD may result in decreased asthma symptoms and improved asthma control

AERD, aspirin exacerbated respiratory disease; NSAID, non-steroidal anti-inflammatory drug; CRS, chronic rhinosinusitis.

**Figure 1:**
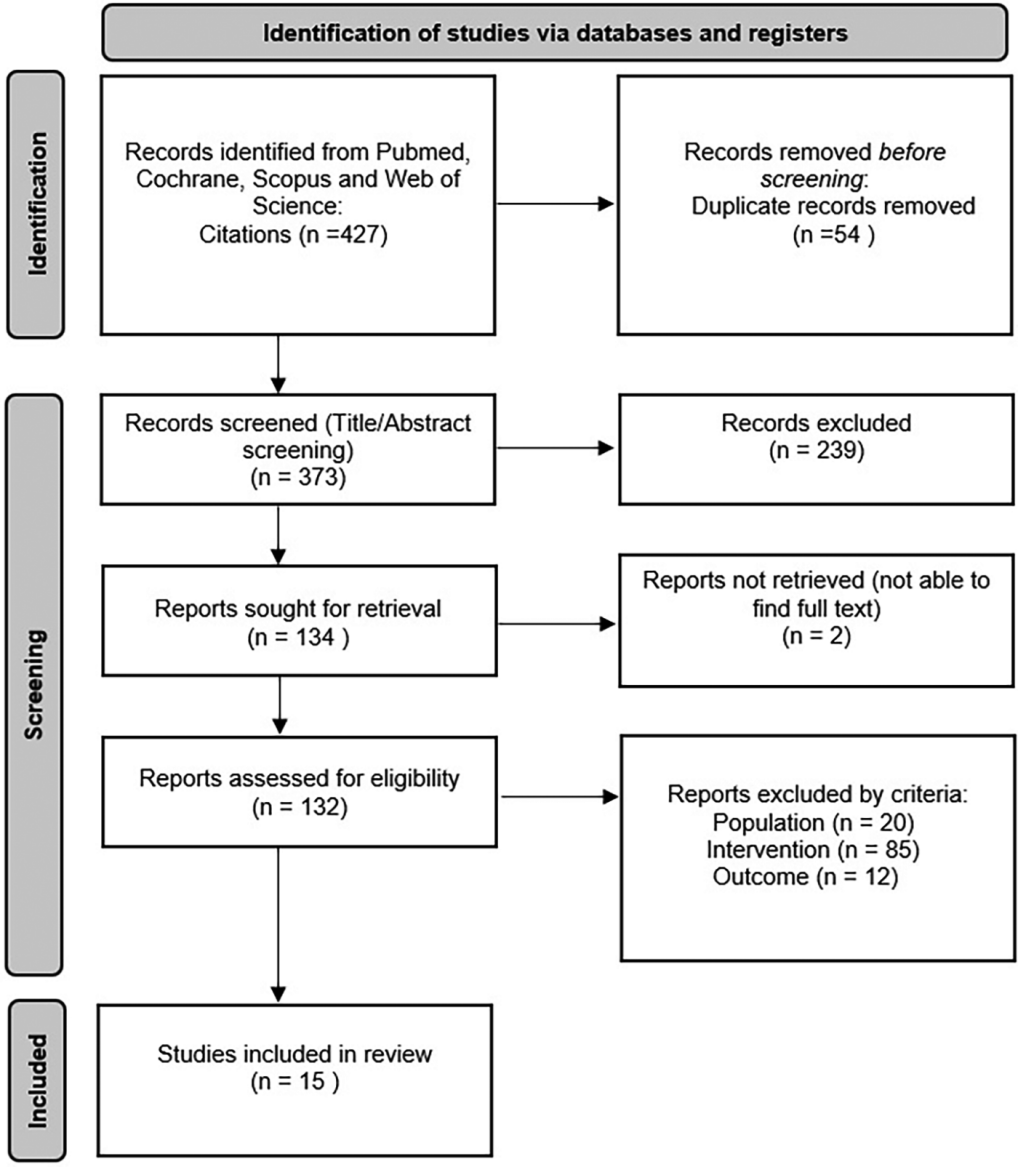
Study search flowchart.

The search strategy identified 427 articles. Once 54 duplicates were removed, all abstracts were screened. Ultimately, 15 papers were selected for review (Figure 1).

The data extraction outcome is shown in Table 5.

**Table 5:** Included studies.

Authors	Reference	Year	Type of study	Adults	N-ERD diagnosis	Intervention	Surgical extension	Follow-up	Recurrence	Results	Biologics	SNOT-22	Effects on QoL	Effects on comorbidities	Relevant conclusions
Kowalski et al.	(13)	2019	Position paper	Yes	Yes	ESS vs. AD + ATAD	Yes	No	No	Yes	No	Yes	Yes	Yes	Yes
Morrissey et al.	(16)	2016	Prospective	Yes	Yes	ESS	Yes	Yes	Yes	Yes	No	Yes	Yes	Yes	Yes
Adappa et al.	(1)	2018	Retrospective	Yes	Yes	ESS + AD + ATAD	Yes	Yes	Yes	Yes	No	Yes	Yes	Yes	Yes
Miglani et al.	(17)	2018	Retrospective	Yes	Yes	ESS	Yes	Yes	Yes	Yes	No	Yes	Yes	No	Yes
Bosso et al.	(9)	2020	Retrospective	Yes	Yes	ESS + AD + ATAD	Yes	Yes	Yes	Yes	No	Yes	Yes	No	Yes
Yoo et al.	(38)	2020	Retrospective	Yes	Yes	ESS	Yes	Yes	Yes	Yes	No	Yes	Yes	No	Yes
Dominas et al.	(11)	2020	Review paper	Yes	Yes	ESS + AD + ATAD	Yes	Yes	Yes	Yes	No	Yes	Yes	Yes	Yes
Piccirillo et al.	(25)	2020	Review paper	Yes	Yes	ESS + AD + ATAD	Yes	Yes	Yes	Yes	No	Yes	Yes	Yes	Yes
Locke et al.	(4)	2020	Retrospective	Yes	Yes	ESS + AD + ATAD vs. ESS alone	Yes	Yes	Yes	Yes	No	Yes	Yes	Yes	Yes
Li et al.	(12)	2019	Review paper	Yes	Yes	ESS + AD + ATAD	Yes	Yes	Yes	Yes	No	Yes	Yes	Yes	Yes
Shah et al.	(2)	2019	Prospective	Yes	Yes	ESS + AD + ATAD	Yes	Yes	Yes	Yes	No	No	No	No	Yes
Jerschow et al.	(46)	2019	Prospective	Yes	Yes	AC + ESS + AD + ATAD	Yes	Yes	Yes	Yes	No	No	Yes	Yes	Yes
Roland et al.	(47)	2020	Retrospective	Yes	Yes	ESS vs. AD + ATAD	No	Yes	Yes	Yes	No	No	Yes	Yes	Yes
Pendolino et al.	(3)	2022	Retrospective	Yes	Yes	ESS	Yes	Yes	No	Yes	No	No	Yes	Yes	Yes
Sweis et al.	(5)	2021	Retrospective	Yes	Yes	ESS + AD + ATAD	Yes	Yes	No	Yes	No	Yes	Yes	Yes	Yes

N-ERD, NSAIDs exacerbated respiratory disease; SNOT-22, sinonasal outcome test-22; QoL, quality of life; ESS, endoscopic sinus surgery; AD, aspirin desensitization; ATAD, aspirin therapy after desensitization; AC, aspirin challenge.

All of the papers (100%) recommended performing ESS in the context of AERD treatment due to the beneficial effect on patient status, and quality of life reflected on indexes as the sinonasal outcomes test (SNOT-22) (25). However, none of them (0%) recommended ESS as single treatment due to the lack of a sustained effect in time. In addition, 100% of the cited papers proposed that additional measures (like AD + ATAD) should be taken.

In Table 6, we show a comparison of ESS alone vs. ESS + AD + ATAD approaches.

**Table 6:** Approaches proposed.

Criteria	ESS	ESS + AD + ATAD
Surgical extension	Full house	Full house
Follow-up	36–66 months	12–42 months
SNOT-22 post-surgery	Immediate and abrupt decrease after surgery	Immediate and abrupt decrease after surgery
Recurrence	2%–26.2%	9.40%
Effect on QoL	Quick improvement immediately after ESS	Quick improvement immediately after ESS
Effect on comorbidities (Represented by CRS and asthma)	Improvement of all the parameters used	Improvement of all the parameters used
Recurrence over time	Improvement sustained between a few months and a year after surgery	Improvement sustained up to 5 years after surgery

ESS, endoscopic sinus surgery; AD, aspirin desensitization; ATAD, aspirin therapy after desensitization; SNOT-22, sinonasal outcome test; QoL, quality of life; CRS, chronic rhinosinusitis.

Regarding the extent of the surgical procedure, almost all of the reviewed studies (93.3%) recommend a full-house approach as a therapeutic measure, without differentiation between groups. The same was found for the SNOT-22 post-operative indexes, where an immediate and abrupt significative decrease was observed in both groups. The mean score before ESS was 47.0, 15.2 for the 1-month post-ESS group, 20.7 for the 1-month post-desensitization group and 22.6 for the 30-month post-desensitization group (1).

Follow-up was extended from 36 to 66 months (average follow-up 51 months) in the ESS group and from 12 to 42 months in the ESS + AD + ATAD group (average follow-up 27 months), while recurrence and the need for reintervention ranged from 2% to 26.2% in the first group and 9.40% in the second.

Eleven out of fifteen papers (73.4%) mentioned an association between ESS and outcomes in patients’ comorbidities, regardless of the approach. All of them agreed on an improvement in all the indices used, including the patient's perception of the disease.

The main difference found was in the time it took to observe a relapse. While in the ESS group this was only a few months after surgery, in the ESS + AD + ATAD group the improvement was maintained up to 3 years after surgery.

## Discussion

There are several pros and cons to be considered before suggesting an option to an individual patient. ESS is performed to remove polyps and alleviate symptoms, such as nasal congestion, runny nose, and facial pressure. It can improve sinus drainage, lower the risk of sinus infection, and improve respiratory function. A reduction in the required medications may result, but the patient should be advised that ESS is very rarely curative and that long term local treatment is necessary. Other considerations should be discussed in advance, such as the associated surgical risks (e.g., bleeding, infection, blindness, CSF leaks and anaesthesia-related problems), the need for revision surgery (due to the associated recurrence of the disease), the recovery time, and the direct and indirect associated costs.

Our review suggests that AD/ATAD probably extends the benefit of ESS in AERD, but there are significant problems with this conclusion.

There was no consensus on how to assess the effectiveness and/or evolution of treatment, nor on the surgical approach used. The SNOT-22 questionnaire and the criteria for the necessity and extent of any revision surgery have been the most commonly used outcomes (26, 27).

We have not found a consistent criterion on the proper timing to initiate AD + ATAD after ESS, nor have we identified a direct relationship between surgical extension and the timing of AD + ATAD. However, in all cases AD + ATAD was initiated between 2 and 6 weeks post-surgery.

The surgical extent proposed and evaluated was variable between studies, ranging from a targeted approach to a full house technique (28). Although there is no international consensus on the extent of the approach required, almost all reviewed studies recommend an aggressive initial surgical approach, such as full house. We conclude that the surgical extension could be explained by the fact that most of the recruited participants were patients with a history of previous ESS.

Patients with AERD who undergo more extensive ESS have been reported to have better outcomes than those who undergo more conservative sinus surgery, with lower recurrence and need for revision rates, and improved quality of life scores (29–31). We consider this is because large quantities of inflammatory tissue have been removed and because post-operative nasal patency facilitates appropriate subsequent pharmacological treatment. In addition, patients with AERD, compared with non-AERD patients, have twice as many sinus surgeries in their history and tend to be younger at the time of their first surgery (3). We consider that this is due to its association with asthma and other comorbidities, its highly eosinophilic nature and that the burden of the disease is greater with respect to CRSwNP alone.

It should be noted that targeted approaches were more common in patients undergoing their first ESS and full house technique was mostly performed in those undergoing a revision surgery. In any case, the extension of the surgical approach will be determined by the extent of the area involved and the experience and criteria of the surgeon (32–34).

Failure rates of standard ESS in these patients are reported to be as high as 90% at 5 years, while rates of revision surgery range from 38% to 89% at 10 years (3, 35).

As follow-up rates were not uniform between studies and, in some cases, very dissimilar, it is not possible to draw associations beyond a three-year postoperative period.

The case for recurrence rates on full house approaches was similar. Many papers did not declare these; however, from those that did we were able to establish a polyp recurrence of 58%. In addition, the need for revision surgery varied between 2% and 26.2%.

All papers described a symptomatic improvement, reflected in a marked decrease in SNOT-22, immediately after surgery (36–38). There was no consensus as to the duration of this therapeutic relief, which ranged from one year for the ESS group to three years for the ESS + AD + ATAD group, after which it tended to decrease and return to baseline (39).

In groups controlling for associated comorbidities, ESS has been shown to contribute to improvements in the severity and frequency of sinonasal and asthmatic symptoms (40–42), radiographic and endoscopic scores, and quality of life after surgery (43–45).

Jerschow et al. (46) found that prior to ESS, the diagnosis of AERD was confirmed in all patients in the study by aspirin tests that elicited hypersensitivity reactions. After ESS, aspirin reactions were less severe in all patients and twelve of twenty-eight patients (43%, *p* < 0.001) had no detectable reaction. This group proposes that the aspirin challenge would have a positive reinforcing effect on the surgical outcome, and the latter on the patient's response to aspirin exposure, justifying the low/zero levels of response seen after surgery.

Roland et al. (47) evaluated patients with AD on a two-months follow-up. Patients who underwent AD experienced a longer time between surgeries compared to patients who did not undergo AD.

Biologics have demonstrated their therapeutic efficacy in patients with AERD and their advent compels us to continue research into their precise indications in the upper and lower airways (13). Our knowledge and consensus on the therapeutic gold standard for these patients is likely to be revised in the short term and they may possibly become the new therapeutic gold standard. However, there is still a good case for initial use of ESS plus ATAD as it is considerably cheaper and very effective in some subjects (5, 48).

## Conclusion

We conclude that while surgery plays a key role in the treatment of patients with AERD, as reflected by significant improvements in quality of life and comorbidities, the combination with postoperative AD + ATAD maintains this improvement in long-term follow-up, also reducing recurrence and revision surgery rates, so that a combined approach should always be considered.
